# Topics of Public Interest

**Published:** 1907-09

**Authors:** 


### TOPICS OF PUBLIC INTEREST.

### The New State Board of Medical Examiners.

**Informal Meeting June 24, 1907—Address of Dr. Andrew S. Draper, Commissioner of Education—Remarks of Dr. Rogers, First Assistant Commissioner, and of Regent Albert Vander Veer, M. D.—Regular Meeting August 1, 1907—Remarks of Dr. Lewi, Secretary—Election of Officers.**

AN informal preliminary meeting of the State Board of Medical Examiners appointed under chapter 344 of the laws of 1907 was held in the rooms of the education department at the capitol, Albany, N. Y., June 24, 1907.

The meeting was called to order at 3 p.m. by Dr. Andrew S. Draper, Commissioner of Education. There were present Dr. William Warren Potter, Buffalo; Dr. William S. Searle, Brooklyn; Dr. Lee H. Smith, Buffalo; Dr. William S. Ely, Rochester; Dr. Eugene Beach, Gloversville; Dr. Floyd M. Crandall, New York City; Dr. Frank W. Adriance, Elmira; Dr. Floyd S. Farnsworth, Plattsburg; Dr. Ralph H. Williams, Rochester, comprising the newly appointed Board of Medical Examiners; also Dr. Howard J. Rogers, First Assistant Commissioner of Education; Dr. Albert Vander Veer of the Board of Regents, and Dr. Maurice J. Lewi, the recently appointed secretary to the State Board of Medical Examiners.

Dr. Draper presented each member of the board with an engrossed certificate of appointment, after which the oath of office was administered by Deputy Secretary of State Whalen.

Dr. Draper in his opening remarks called attention to the fact that this first meeting must necessarily be an informal one inasmuch as under the terms of the statute the board had no legal existence until August 1, 1907. He stated, however, that there were several reasons for calling an earlier conference: first, that the members might become acquainted with one another; second, that there might be some understanding of the routine necessary in carrying out the purposes of the law. He mentioned briefly the considerations which had led to the appointment of the board, from the department point of view and from the legislative point of view, and spoke of the plan upon which it was hoped the work of the board would be prosecuted. "Something in this direction," he said, "had come to be inevitable. The doctors had tired out the legislature and the legislature had made up its mind that it would do something drastic some day to put an end to the eternal fuss that was coming up from the doctors. The fact had come to be pretty clear that either there had to be more examining boards or less, and that it was hardly feasible to go on indefinitely multiplying examining boards; also that the state to the fullest measure possible ought to avoid entanglements with the different schools or representatives of the different schools in medicine.

It came to be pretty clearly understood by the department some time ago that the functions of a state examining board,—the protection of the public and the integrity of medical practice,—would be very well met when the state should see to it that every candidate for medical licensure should be possessed of moral character and of scientific attainments, which should qualify him for dealing with the salient hurts and ailments to which humanity is subject. In any event, for weal or woe, the state has determined to try the experiment of barring all candidates for medical practice who do not show moral character sufficient to merit the general commendation of the state, and scientific knowledge which is adequate to the protection of the people of the state. The association of the various members of the board with different schools of medicine is a matter of indifference to the department.

This board has been made up by the Board of Regents after careful, painstaking discussion of the whole subject. It is no small compliment to the physician in practice to have received an appointment upon this initial board of medical examiners. I congratulate the department and the board upon the selection of Dr. Lewi as the secretary of the board. He has had large experience in medical examinations work and we shall feel very much safer with the knowledge that he is to have charge of the details, than we would if any stranger to medical examinations work were to assume the charge of these endless details which are associated with the protection of the medical profession, and the examination of those who are coming along all the time seeking admission to that profession.

We ought all to work together without fear and without favor, pursuant to a determination to agree upon policies which will be for the uplifting of medical practice in this state; which will hold out a fair and square deal to all, without reference to their previous affiliation or their professional beliefs. There is no profession for which I have so complete respect when it is represented by men of integrity and reasonable learning and considerable experience. On the other hand there is no profession so peculiarly subject to the barnacles of charlatanism of every conceivable kind, and there is no man for whom I have such complete contempt as for the man who lacks in learning and lacks in moral sense, yet masquerades under the garb of this profession.

These appointments have been made with the expectation that all the members of this board will cooperate to execute the law in all good spirit and purpose, to the end that the practice of medicine, so far as any act of this board can make it so, shall stand upon a solid and substantial moral and scientific footing."

Dr. Ely in responding on behalf of the board said: "I am sure all the members of the board have listened to these remarks with a due appreciation of what has been said, and that we have all come together to work in the interests of this bill. While there has been a great deal of discussion in the papers with respect to this matter, we are going to work out the problem to the great credit of this state. There will be no sectarian feeling among us you may be sure. We shall all join together heartily and make the success of this measure our most profound endeavor.

As one who has known the secretary of the board and the work he has done in the past, I feel that we are under great obligations to the regents for having reappointed Dr. Lewi as secretary, and I am sure that he will give to the new members of the board all the help and assistance they will need in the beginning of the work."

Dr. Vander Veer, on behalf of the regents, congratulated the board on the full attendance at this initial meeting and expressed the hope that they would work together harmoniously so that the public would look to the board as working for the good of the profession at large.

Dr. Rogers stated that the main reason for calling the board together at this time was for the purpose of securing questions for the September examination, which it was necessary to arrange for at once, with the additional object of having the members take the oath of office and get acquainted with one another.

Dr. Lewi explained the manner of making up the question papers and the necessity for the early submission of questions, and asked that 25 questions in each topic be submitted by July 1, for the September examination.

REGULAR MEETING AUGUST 1, 1907.

A regular meeting of the State Board of Medical Examiners was held at the capitol, Albany, August 1, 1907.

Dr. Lewi, in calling the meeting to order at 12 o'clock noon, spoke informally as follows touching his appointment as secretary of the board:

Gentlemen: The kindly words of praise spoken by the Commissioner of Education at the preliminary conference of this board, together with the equally pleasant response to the commissioner made by Examiner Ely, call for my respectful expression of thanks. I trust in my position to merit a continuance of the approval of those who have been good enough to honor me with their confidence. I believe a golden opportunity presents itself to the members of this board. Knowing no creed in medicine, having no internal battles to wage, we should present a united front as servants of the state and should thus be able to do yeoman service in its behalf. We surely have come to a time in medicine when the study of the subject in all its ramifications should have for its prime purpose the prevention rather than the cure of disease. If you as a board of examiners can so fashion your work as to instil into the minds of the medical teaching profession the thought that the thorough study of sanitation, hygiene, physiologic chemistry, bacteriology and pathology are the essentials for the succesful physician, advances along these lines will be made by the teaching bodies and the interests of the state will be splendidly served because thereof.

A number of years ago, I suggested that the regents of the University of the State of New York create the title of Doctor of Sanitation, and that, through a board of examiners, examinations be conducted for passing upon the qualifications of such as would earn this distinction. I am still of the opinion that such a procedure would be wise, and that in this connection the legislature of the state of New York should be asked to enact a law which would bring it to pass that on and after a date, not too remote, no person could serve as health officer unless he be a doctor of sanitation. If in the state of New York there were today a scientific and experienced hygienist in every hamlet, village, and city, there is no question but that diseases which are of daily occurrence could and would be prevented. This suggestion is made without any disrespect to the existing health officers of the state, but with a view solely to placing on guard at every vantage point men who will prove competent to serve the state as equipped sanitarians. I am pleased to state that Dr. Rogers is at one with me in this suggestion.

I trust that the work of the year will be congenial to all the members of the board, and I herewith notify you severally and collectively that my services are at your disposal at any and all times.

Moved by Dr. Smith and adopted, that a committee be appointed to consider the suggestion made by Dr. Lewi and to report at a future meeting.

The chair announced as this committee Drs. Smith, Beach, and Ely.

Dr. Adriance offered the following resolution, which was unanimously adopted.

Whereas, at an informal meeting of the State Board of Medical Examiners held in the office of the education department, June 24, 1907, a tenative assignment of topics was made to the various members of the board by the education department, which division of topics seems to have worked harmoniously and to the satisfaction of the board of regents and the board of medical examiners,

Resolved, That this assignment of topics be confirmed for the academic year beginning August 1, 1907, and that the subjects and the examiners therein be as follows:

| | |
|---|---|
| Anatomy | Dr. Ely |
| Physiology | Dr. Williams |
| Hygiene and Sanitation | Dr. Beach |
| Chemistry | Dr. Farnsworth |
| Surgery | Dr. Crandall |
| Obstetrics and Gynecology | Dr. Potter |
| Pathology | Dr. Smith |
| Bacteriology | Dr. Adriance |
| Diagnosis | Dr. Searle |

Dr. Crandall offered the following resolution which was unanimously adopted:

Whereas, At an informal meeting of the State Board of Medical Examiners held in the education department, June 24, 1907, a question committee consisting of Dr. Smith, of Buffalo, as chairman, Dr. Potter, Dr. Adriance and Dr. Williams was appointed,

*Resolved*, That this action be confirmed and that the question committee be continued for the academic year beginning August 1, 1907, as follows: Dr. Smith, Dr. Potter, Dr. Adriance, Dr. Williams.

The secretary read a communication from Dr. Howard J. Rogers, first assistant commissioner of education, transmitting for the consideration of the State Board of Medical Examiners, charges of the Medical Society of the County of Erie relating to the registration of William W. Turver, now serving a sentence in Auburn state prison, asking that it be annulled. The papers, including a certified copy of the judgment of the court, were referred to a committee for investigation, under subdivision *e*, section 11, of chapter 344, laws of 1907.

The secretary submitted a draft of rules and regulations for the government of the board, which was unanimously adopted.

Dr. Potter read a letter from the council on medical education of the American Medical Association asking that a delegate from New York be appointed to represent the board at the next conference.

Moved and carried that Dr. Potter be appointed a delegate to represent the New York State Board of medical examiners at the next conference on medical education of the American Medical Association.

The election of officers for the academic year beginning August first, resulted as follows: president, William Warren Potter, Buffalo; vice-president, William S. Searle, Brooklyn.

Adjournment, sine die, was then taken.

---

### To Prevent Substitution.

GOVERNOR HUGHES has signed the amendment to section 401 of the penal code of the state of New York, which, it is believed, will put an end, practically at least, to the pernicious practice of substitution of physicians' prescriptions on the part of druggists. The new law reads as follows:

Section 401. Any person, who, in putting up any drug, medicine or food or preparation used in medical practice, or making up any prescription, or filling any order for drugs, medicines, food or preparation puts any untrue label, stamp or other designation of contents upon any box, bottle or other package containing a drug, medicine, food or preparation used in medical practice, or substitutes or dispenses a different article for or in lieu of any article prescribed, ordered or demanded, or puts up a greater or less quantity of any ingredient specified in any such prescription, order or demand than that prescribed, ordered or demanded, or otherwise deviates from the terms of the prescription, order or demand by substituting one drug for another, is guilty of a misdemeanor; provided, however, that, except in the case of physicians' prescriptions, nothing herein contained shall be deemed or construed to prevent or impair or in any manner affect the right of an apothecary, druggist, pharmacist or other person to recommend the purchase of an article other than that ordered, required or demanded, but of a similar nature, or to sell such other article in place or in lieu of an article ordered, required or demanded, with the knowledge and consent of the purchaser. Upon a second conviction for a violation of this section the offender must be sentenced to imprisonment, for a term of not less than ten days nor more than one year, and to the payment of a fine of not less than ten dollars nor more than five hundred dollars. The third conviction of a violation of any of the provisions of this section, in addition to rendering the offender liable to the penalty prescribed by law for a misdemeanor, shall forfeit any right which he may possess under the law of this state at the time of such conviction, to engage as proprietor, agent, employee or otherwise, in the business of an apothecary, pharmacist or druggist, or to compound, prepare or dispense prescriptions or orders for drugs, medicines or foods or preparations used in medical practice; and the offender shall be by reason of such conviction disqualified from engaging in any such business as proprietor, agent, employee or otherwise, or compounding, preparing or dispensing medical prescriptions or orders for drugs, medicines or foods or preparations used in medical practice.

SECTION 402. This act shall not affect or impair any liability, penalty or punishment under the provisions of section four hundred and one as the same existed prior to the time this act takes effect, but the same may be enforced, prosecuted or inflicted as fully and to the same extent as though this act had not been passed; and all actions civil or criminal instituted under or by virtue of said section as the same existed prior to the passage of this act, and pending immediately prior to the taking effect hereof, may be prosecuted and defended to final effect in the same manner as though this act had not been passed.

SECTION 403. This act shall take effect September first, nineteen hundred and seven.

---

### The Secretary of a County Medical Society.

BY J. B. DONALDSON, M.D.

Secretary of the Washington County (Pennsylvania) Medical Society.

[*Pennsylvania Medical Journal, December, 1906.*]

SHOW me your secretary, and I will tell you whether your society is a success or a drag. If he is prompt, active, and energetic, the society is bound to be a good one. I firmly believe the society is just what he sees fit to make it. Let me qualify this statement; I am speaking of the rural societies, not of the counties that contain large cities where they have a committee on program, and the like. I do not want to appear egotistical, and hope you will pardon me for alluding so frequently to my own society, but I know what has been done there, and know the material to do it with.

This is an age of organisation, and the profession has awakened to the necessity of doing something along these lines, but the average doctor is a mighty hard man to get interested in anything, especially in a scheme that he thinks will take his time. And, again, he feels that, if Dr. So-and-So is going to run the society, he "won't play." I often have men tell me, "Well, if Dr. Blank is a specimen of your society, I want none of it in mine." Such men are rapidly becoming extinct, thank the Lord, and in a few years they will all be dead or have joined the ranks of the irregulars, where they belong. They remind me of Sam Jones's lecture in which he rips the hypocrites up the back in about this language: "You old Presbyterian brother, out there, that won't speak to your neighbor when you meet him on the road, why, the only reason you ain't in hell is because you ain't dead." And it is so. The doctor who won't speak to his confrere is rapidly becoming extinct. The young men are not trained along these lines. Why, you and I were taught to hate a homeopath like a snake. Our professors were men of these strong likes and dislikes, and did *us* much harm by teaching it. It takes a broad man almost a lifetime to get such stuff out of his head.

The secretary must be a diplomat, and a busy man. To him will almost all grievances come, and it is up to him to pour oil on the troubled waters. You can generally bank on the average kicker not being very familiar with the rules of his society, and he generally writes the secretary for instruction, or for a copy of the by-laws, for he is sure to have mislaid his copy. Answer his letter promptly, but don't hurry up the by-laws if his point is too well taken. A little time soothes a whole lot, and he won't be quite so mad next week.

As to answering letters, a secretary should *never* allow a letter to remain over night unanswered. It is simply damnable the way the average doctor treats his correspondence. He pays about as much attention to it as he does to his prayers. He will not answer at all if it is possible to get out of it. Every secretary here will bear me out in this, I know. But for a secretary to be careless in this respect is simply unpardonable, no matter how trivial the request may appear to you. Let your work be systematic in every respect. Your notices must always come out on time. They should be neat and attractive in appearance. *Don't* send out postal card notices. You don't use postal cards in your own correspondence, and why should your society? We use a sheet, that we have printed for us at the beginning of the fiscal year, with quite a lot of information on it, as a sort of letter head. These are printed on one side only, in bulk, and are used for the programs each meeting, leaving a space for writing a few lines if needed. The difference in cost is trivial, and well worth the money. Neater designs will occur to many of you, but I think the idea a good one, and I have been flattered to have men from our own and other states thank me for the idea, which they had adopted. The practitioner is a busy man. His mind is not on society work, and you must keep him posted. You can do it in this way. Tell him every month when the state society and the American Medical Association meet and where. He won't mind it from one month to the next, but keep on telling him. Tell him who the officers are, how many members you have, when you were organised, and such other information as may occur to you that every medical man should know. Keep before him all the time how a man may become a member of the society and what he gains by so doing. By and by he will absorb these things and be proud to tell his folks.

For your secretary always pick a busy man. It is a mistake to say, Dr. Blank does not have much to do, and will make a good secretary. The busy fellow is the chap you want. He does not get weary half as soon as the idle fellow, who generally does not have time to answer his letters or pay his bills. Pick a young man if you can with the requirements, but don't turn him down if he happens to be fifty and busy.

Once a society gets a good secretary keep him at it until he begins to flag, then turn him down. Many a good society is kept in the mediocrity rank because they don't like to hurt the old secretary's feelings. It is a mistake! Fellow secretaries, as soon as you feel that you can't keep up the pace, resign! Keep up the interest in your meetings by having a strange speaker every time. The element of curiosity helps bring some. But don't neglect bringing out the young bashful talent of your own men. Make them and everybody else think the program depends largely on them. It is easy if you get them going once. Every society has men that will surprise you if by a little urging you get them interested. Don't have too much on your program. "Enough is as good as a feast," and, if your men go away tired, they don't carry home with them the feeling of satisfaction that you want. If the county is a large one it may be of advantage to have one or two meetings each year in some other town than the regular meeting place, but our experience is that it does not do much good. Men get in the habit of going to a certain place and will not be pleased elsewhere.

Have an attractive place to meet. We have furnished for us by the county, in the court house, a beautiful room, adorned with portraits of our ex-presidents and noted dead.

As to the number of meetings each year, that can not be arbitrarily arranged. We meet bimonthly now, having increased the meetings from three to six per year.

A secretary must of course never miss a meeting. Nothing short of real sickness in his family should justify his absence.

You run the risk, in taking the initiative in almost every movement that your society makes, of having the knockers say, "We have a little too much Donaldson (or whatever your name is) in our society." The only remedy for this is, like Davy Crocket, to "be sure you are right, then go ahead." The knocker you always have with you, and he often is productive of much good in helping to keep you in bounds. Make an ally of that chap as fast as you can. Make him *particeps criminis*. He is often a good fellow in disguise. If I were making a scientific diagnosis of his case, as a rule I would say he is suffering from dementia, paranoia, or more frequently dementia praecox.

Never allow your minutes to remain unwritten twenty-four hours after a meeting. I have known minutes of county medical societies to remain unwritten for over ten years. Imagine the consternation of that secretary when called to produce the minute book; for the knocker finally got up the courage to demand it of a superannuated secretary, who should have been retired after his tenth year. You say, "Why, you expect a secretary to be immaculate!" and "Such men are impossible!" Not at all. First of all, your heart must be in the work, or don't take it. If you accept the office, put into it your whole heart, mind, and energy, and success is bound to follow. Put into it the same kind of work and brains you do in your business. Don't flag at any one point; if you do, you will make a failure. It is a pleasure to feel that you are the instrument of doing much good for your fellow doctors. The greatest pleasure of my life is to be in the society of the doctors of my community and state. About all the vacations I ever get are at the meetings of this and kindred societies, where I renew old acquaintances and form new ones that are a joy to me. And why shouldn't I enjoy these? They are the best fellows on earth; all you have to do is know them.

A secretary should never miss a meeting of his state or the national society. If possible be one of the representatives in its executive body. You will learn much as to how to deal with men. Belong to all the medical societies you can get into; attend and take part in them.

There is much that might be said as to the duties of a secretary, but it would be impossible to cover the ground in a five minute paper. To sum up the requisites of a good secretary, let me say briefly, and in conclusion, as the preachers say, use your brains, and if you don't have many, work what you have to the limit, and you will get results.

Anything will do for a president of a society, but not so as to the secretary.

---

A. Ernest Gallant of New York believes that weight of clothing and compression at the waist line at and after puberty have an important effect on girls in producing movable kidneys. The author has reviewed fifty cases and made careful measurements and finds abnormalities in the length of the trunk, as shown by the distance between the suprasternal notch and upper border of the symphisis pubis, and deviation from the normal relation between the circumference of the waist and hips at the trochanters. There is hollowing of the epigastrium and bulging of the hypogastrium. A respiratory rise and fall of the greater curvature of the stomach may be seen in thin-walled subjects. Displacement occurs frequently after child-birth and should be remedied by a firm abdominal binder and exercises while in bed. Prolapse of the colon and stomach are generally associated with that of the kidney. These conditions are not remedied by operation. A properly shaped corset, put on before rising, in a semi-opisthotonos position, makes the patient comfortable and prevents complications.—*Medical Record,* July 27, 1902.

---

Chas. A. Rosenwasser of Newark, N. J., speaks highly of the value of apomorphine as a sedative and hypnotic in cases of acute alcoholism. It occurs in two forms, the amporphous and the crystalline, of which the latter is preferable. The usual dosage is from 1-30 to 1-10 grain hypodermically. A very few minutes after administering an emetic dose by hypodermic injection, vomiting occurs, due to the action of the drug upon the vomiting center in the medulla. Just before vomiting the pulse is weakened and increased in frequency, and after vomiting ceases it becomes stronger and slower, often stronger and slower than it was before the injection was given. Vomiting is preceded by salivation and slight nausea. It may occur only once, or be repeated several times. Very soon after the vomiting subsides, a matter of a few minutes in most cases, the patient falls into an apparently natural sleep, and may sleep for from two to eight hours, awakening refreshed, sober, and rational in most cases. It is not necessary, however, to give the emetic dose in order to obtain the hypnotic effect, and in many cases 1-30 grain will induce sleep.—*Medical Record,* July 27, 1907.

